# The Additional Value of Somatostatin Receptor Positron Emission Computed Tomography ([^68^Ga]Ga-DOTATOC PET/CT) Compared with Magnetic Resonance Imaging of the Head and Neck Region in Paraganglioma Patients: A Pilot Study

**DOI:** 10.3390/cancers16050986

**Published:** 2024-02-28

**Authors:** Carolijn J. M. de Bresser, Bart-Jeroen Petri, Arthur J. A. T. Braat, Bart de Keizer, Mark J. C. van Treijen, Jan Willem Dankbaar, Frank A. Pameijer, Marius G. J. Kok, Mischa de Ridder, Bernadette P. M. van Nesselrooij, Remco de Bree, Gert J. de Borst, Johannes A. Rijken

**Affiliations:** 1 Department of Vascular Surgery, University Medical Center Utrecht, Heidelberglaan 100, 3584 CX Utrecht, The Netherlands; 2 Department of Radiology and Nuclear Medicine, University Medical Center Utrecht, 3584 CX Utrecht, The Netherlands; 3 Department of Nuclear Medicine, Netherlands Cancer Institute, 1066 CX Amsterdam, The Netherlands; 4 Department of Endocrine Oncology, University Medical Center Utrecht, 3584 CX Utrecht, The Netherlands; 5 Department of Radiology, University Medical Center Utrecht, 3584 CX Utrecht, The Netherlands; 6 Department of Radiology, Medical Spectrum Twente, 7512 KZ Enschede, The Netherlands; 7 Department of Radiotherapy, University Medical Center Utrecht, 3584 CX Utrecht, The Netherlands; 8 Department of Clinical Genetics, University Medical Center Utrecht, 3584 CX Utrecht, The Netherlands; 9 Department of Head and Neck Surgical Oncology, University Medical Center Utrecht, 3584 CX Utrecht, The Netherlands

**Keywords:** head and neck paraganglioma, [^68^Ga]Ga-DOTATOC PET/CT, MRI

## Abstract

**Simple Summary:**

All patients suspected of having head and neck paragangliomas (HNPGLs) undergo magnetic resonance imaging (MRI) or computed tomography (CT) of the head and neck area as the standard imaging according to the Dutch guidelines. However, a relatively new imaging modality, [^68^Ga]Ga-DOTATOC PET/CT, has shown promising results in the detection of HNPGLs. In this pilot study, we aimed to compare the results of this new modality with the standard imaging. We compared both techniques in 25 patients at the University Medical Center Utrecht, The Netherlands. Twenty-eight percent of the patients had different outcomes between both modalities. In all cases, additional HNPGLs were identified using the [^68^Ga]Ga-DOTATOC PET/CT, suggesting the need for further prospective research to validate this finding. If confirmed, this modality could potentially be implemented as the standard of care for HNPGL detection in germline variant carriers.

**Abstract:**

The Dutch guideline for patients suspected of head and neck paragangliomas (HNPGLs) recommends magnetic resonance imaging (MRI) and/or computed tomography (CT) of the head and neck area. Additionally, it suggests considering additional nuclear imaging. The aim of this study was to evaluate the outcomes of [^68^Ga]Ga-DOTATOC PET/CT compared to MRI in patients with suspected HNPGLs and carriers of genetic variations. Methods: In this single-center pilot study, retrospective data were obtained from consecutive patients between 2016 and 2023. Both MRI and [^68^Ga]Ga-DOTATOC PET/CT were performed within 12 months. The primary outcome was the location of HNPGLs. Results: A total of 25 consecutive patients were included, and 7 patients (28.0%, *p* = 0.5) showed differences between the imaging modalities, of whom 5 patients had unexpected localizations with additional uptake by somatostatin receptors (SSTR) on the [^68^Ga]Ga-DOTATOC PET/CT. Conclusions: The authors recommend performing baseline imaging with [^68^Ga]Ga-DOTATOC PET/CT (if available) in variant carriers and using MRI/CT for follow-up according to the regional protocol, thereby shifting the gold standard for baseline imaging from MRI/CT to [^68^Ga]Ga-DOTATOC PET/CT.

## 1. Introduction

The management of head and neck paragangliomas (HNPGLs) is challenging due to their usually benign, slow-growing, and highly vascularized character. The disadvantages of surgery or radiotherapy often outweigh the limited risks that can arise in the natural course of HNPGLs. Management options for HNPGL patients include active surveillance, radiotherapy, surgical resection, or combinations. In most cases, a wait-and-scan policy is adopted, as HNPGLs are slow-growing and located in the complex anatomical area of the head and neck in close proximity to cranial nerves [1]. A significant proportion of patients with HNPGLs have genetic variations in the succinate dehydrogenase (SDH) genes, which increase their risk of developing single or multiple paragangliomas throughout life [2,3]. 

The Dutch Guideline for Head and Neck Paragangliomas, based upon expert opinions, recommends a computed tomography (CT) or magnetic resonance imaging (MRI) of the head and neck (h-nMRI), cranial nerve function examination, the measurement of metanephrines, genetic counseling, and DNA testing for the screening of possible HNPGLs [4]. An additional CT scan of the mastoid is performed if a jugulotympanic paraganglioma (JTPGL) is suspected. Whole-body-screening MRI is advised for carriers of variants in the SDH subunit B gene and in cases of catecholamine hypersecretion [5]. Carriers of genetic variations are screened from 18 years (10 in SDHB) to detect paragangliomas at the earliest stage for the optimal timing of intervention [6,7].

Paragangliomas (PGLs) and pheochromocytomas (PHEOs) overexpress somatostatin receptors (SSTRs), generally subtype SSTR_2_ [8,9,10]. SSTR-targeted radiopharmaceuticals are widely available for diagnostic imaging with positron emission tomography (PET), also known as [^68^Ga]Ga-DOTATOC PET/CT, binding to all SSTR subtypes, with a preference for SSTR_2a_ [11,12]. [^68^Ga]Ga-DOTATOC PET/CT has shown a higher lesion-based detection rate (LBD) with 98.6% [95% confidence interval (CI), 96.5–99.5%] compared to CT/MRI 85.8% [95% CI, 81.3–89.4%] [13]. CT and MRI provide excellent anatomical detail and a high sensitivity up to 100%, but lack specificity in distinguishing between PGLs and other lesions [14]. The reported pooled sensitivity of [^68^Ga]Ga-DOTATOC PET/CT was 93% [95% CI, 89–96%], and it had a pooled specificity of 85% [95% CI, 74–93%], suggesting this imaging technique as a first-line modality for the primary staging of paragangliomas in daily practice [15,16,17]. This modality is not a standard of care according to the Dutch guidelines, but suggested as an additional scanning option for sympathetic PGLs, suspected metastases, or cases with high HNPGL suspicion [4].

This pilot study aims to evaluate the added value of [^68^Ga]Ga-DOTATOC PET/CT to conventional h-nMRI in patients with known HNPGLs or carriers of genetic variations causative for HNPGLs, focusing on the head and neck area.

## 2. Materials and Methods

In this single-center pilot study, data were obtained from patients visiting the PGL outpatient clinic (a combined clinic of vascular surgeon, otolaryngologist, endocrine oncologist, and clinical geneticist) of the University Medical Center Utrecht, a tertiary referral center with expertise in HNPGLs, between 2016 and 2023. All patients had at least one HNPGL or a genetic variation associated with the development of PGLs. All patients were included in the Head and Neck Paraganglioma Registry (HNPGLR), approved by the UMC Utrecht medical ethics committee (ID 22-008).

Relevant patient characteristics were retrospectively collected. The diagnosis of HNPGL was based on imaging and was compliant with national and international guidelines [4,18]. All HNPGL patients were offered genetic counselling, and variant carrier status was based on minimal DNA testing for *SDHx* and *MAX*. Patients suspected of pheochromocytoma were also tested for *RET*, *VHL*, *FH*, *MDH2*, and *TMEM127*. Annual biochemical screening included physical examination and the measurement of plasma free (nor) metanephrines (NM/M) and 3-methoxytyramine (3-MT). MRI scanning was performed on all patients. Local MRI protocols differ between genetic variant carriers and patients with (suspected) HNPGLs. At diagnosis and at least every three years afterwards, whole body MRI (wbMRI) was performed in genetic variation carriers without known manifestations. The wbMRI protocol includes a T2 weighted sequence with a fat suppression of the head and neck. Patients with, or suspected of, HNPGLs underwent a dedicated MRI of the head-and-neck area (h-nMRI) consisting of T1- and T2 weighed images, gadolinium-enhanced images, and/or magnetic resonance angiography (MRA). Local protocols can be found in Appendix A. All h-nMRI imaging was performed on Achieva or Ingenia 1.5T or 3T scanners (Philips, Best, The Netherlands). In patients who underwent wbMRI using 1.5T–3T MRI (Achieva or Ingenia), only the head and neck outcomes were used. MRI outcomes were triple-blinded interpreted by radiologists with expertise in HNPGLs. 

In this study, [^68^Ga]Ga-DOTATOC PET/CT was performed in addition to MRI. [^68^Ga]Ga-DOTATOC PET/CT was obtained via the administration of 1.5–2.0 MBq/kg ^68^Ga-DOTATOC, and after an incubation period of approximately 45 min, a whole body (vertex to groin) PET (5 min/bed position; 4 iterations, 21 subsites) with low dose-CT (120 kV and 30 mAs) was acquired (Biograph mCT, Siemens, Erlangen, Germany). Maximum standardized uptake values (SUVs) were measured for all SSTR_2_ positive lesions. For the SUV calculation, a body-weight-corrected formulation was used. PET/CT images were reconstructed according to EARL 2.0. The [^68^Ga]Ga-DOTATOC PET/CT and wbMRI/h-nMRI were performed within 12 months. 

The clinical reporting of this modality was performed double-blinded by two nuclear radiologists with expertise in HNPGLs. Any conflict between experts was solved by discussion until interobserver agreement was reached. In case a HNPGL was resected within the 12-month time frame of both imaging modalities, the patient was excluded from further analyses. 

### Statistics

The definition of a detected lesion was based on the clinical report of both modalities, and retrospectively compared. As a biopsy of lesions is not a standard of care in PGL, available histopathology (i.e., biopsies or surgical resection specimens) were used to validate the findings of h-nMRI and/or [^68^Ga]Ga-DOTATOC PET/CT. In absence of histopathological confirmation, follow-up imaging was used to confirm lesion detection. 

The sensitivities of [^68^Ga]Ga-DOTATOC PET/CT and MRI were compared using a paired *t*-test. Lesion detection was defined based on the clinical reports of both modalities. Histopathology, biopsies, surgical resection specimens, or follow-up imaging were used to validate the findings of MRI and/or [^68^Ga]Ga-DOTATOC PET/CT. The odds ratio was used to assess the association between MRI and [^68^Ga]Ga-DOTATOC PET/CT. Statistical significance was considered at *p* < 0.05.

## 3. Results

### Clinical Characteristics

Twenty-five patients with known or suspected HNPGLs, eleven males (44.0%) and fourteen females (56.0%), underwent both wbMRI/h-nMRI and [^68^Ga]Ga-DOTATOC PET/CT. The clinical characteristics are depicted in Table 1. The mean age was 46.9 years (95% CI, 29.7–64.1 years) and ranged from 21 to 80 years. Twenty-four patients carried a genetic variant (96.0%) in genes coding for SDHA (*n* = 2, 8.0%), SDHB (*n* = 9, 36.0%), SDHC (*n* = 1, 4.0%), and SDHD (*n* = 12, 48.0%). Five patients (*n* = 5, 20.0%) showed elevated catecholamine production. Sixteen patients carried at least one HNPGL and nine patients were variant carriers without HNPGLs. Eleven patients (44.0%) obtained the [^68^Ga]Ga-DOTATOC PET/CT at baseline and fourteen patients (56.0%) obtained the [^68^Ga]Ga-DOTATOC PET/CT during follow-up. 

**Figure 1:**
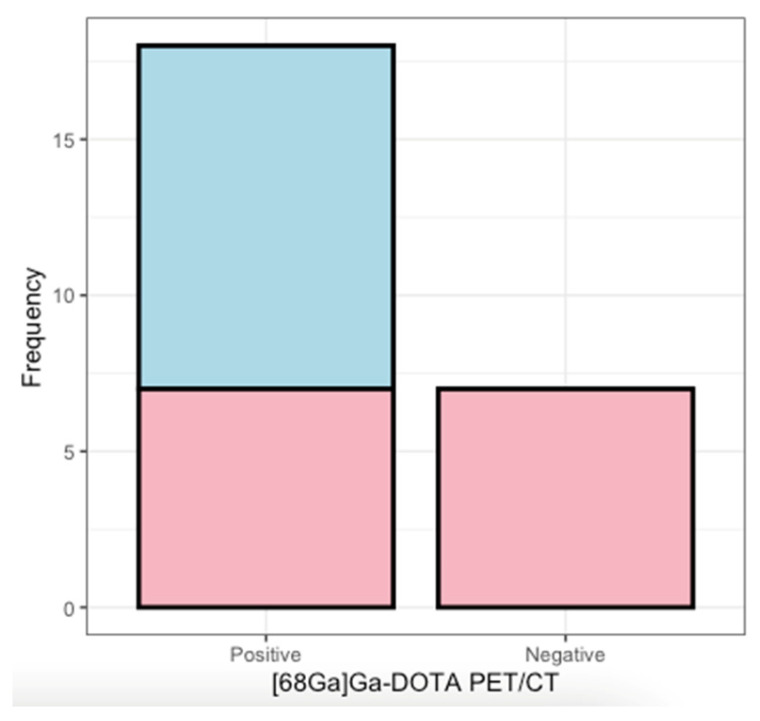
Visualization of the outcomes of the MRI (blue) and [^68^Ga]Ga-DOTATOC PET/CT (red). The *x*-axis depicts the outcomes of the [^68^Ga]Ga-DOTATOC PET/CT with the frequency on the *y*-axis. In 18 patients, the [^68^Ga]Ga-DOTATOC PET/CT shows SSTR_2_ positive lesions. In 11/18, the MRI shows the same lesions, but in 7 cases, the MRI does not show the same lesions. The [^68^Ga]Ga-DOTATOC PET/CT is not showing any SSTR_2_ uptake in seven cases in which the MRI also does not show any lesions. [^68^Ga]Ga-DOTATOC PET/CT = somatostatin receptor positron emission computed tomography; MRI = magnetic resonance imaging; SSTR_2_ = somatostatin receptor type 2.

Table 2 shows all outcomes of patients undergoing wbMRI/h-nMRI and [^68^Ga]Ga-DOTATOC PET/CT. Eighteen patients (*n* = 18/25, 72.0%) showed no difference between the outcomes of MRI and [^68^Ga]Ga-DOTATOC PET/CT. In seven patients (*n* = 7/25, 28.0%, *p* = 0.5, OR = 5.5 (95% CI, −64.4–75.4)), a difference was reported between the modalities. Five patients (*n* = 5/7 (71%)) showed unexpected additional SSTR_2_ uptake in the head and neck area on [^68^Ga]Ga-DOTATOC PET/CT. A total of 7 lesions were discordant by the MRI. Jugulotympanic lesions were predominantly missed (*n* = 5/7, 71.4%), alongside carotid body paraganglioma (CB PGL) (*n* = 1/7, 14.3%), and a vagal paraganglioma (VPGL) (*n* = 1/7, 14.3%), all in patients carrying an SDHD-variation. In two subjects, both imaging techniques identified an equivalent number of PGLs on identical sides, although discrepancies arose in the reported observed locations between nuclear and radiological assessments. Figure 1 provides a graphical overview of similarities and discrepancies between both modalities. In Patient 3 (*n* = 1/25, 2.8%), the results of the additional [^68^Ga]Ga-DOTATOC PET/CT led to a change in management, from initial surgical resection to wait-and-scan. 

Ten patients (*n* = 10/25, 40.0%) underwent eight surgical resections of the paraganglioma and two biopsies. Nine histopathology reports showed paraganglioma tissue. One patient underwent surgical resection of a left-sided CB PGL in a different hospital, and no histopathology was available.

## 4. Discussion

In this study, we conducted a comparative assessment of whole body [^68^Ga]Ga-DOTATOC PET/CT and wbMRI/h-nMRI in a cohort of 25 patients diagnosed with HNPGLs or a genetic variation suspected of causing HNPGLs. We found that the outcome of the [^68^Ga]Ga-DOTATOC PET/CT compared to the wbMRI/h-nMRI was different in 28.0% of the patients, either revealing additional SSTR_2_ positive lesions or distinguishing between MRI suspected lesions without any SSTR_2_ uptake. Additionally, the [^68^Ga]Ga-DOTATOC PET/CT detected more lesions compared to wbMRI/h-nMRI (*p* = 0.5, OR = 5.5 (95% CI, −64.4–75.4)). A possible explanation may be that the lesions detected only by [^68^Ga]Ga-DOTATOC PET/CT are relatively small and have a relatively low maximum SUV compared to lesions detected on both modalities. However, probably due to the relatively low sample size, no statistical significance was found between the two techniques. 

Jugulotympanic paragangliomas (JT PGLs) were most often missed on h-nMRI/wbMRI (*n* = 5/7, 71.4%) as CT is the golden standard for diagnosing lesions in close proximity to the skull base [19]. A CT of the mastoid and middle ear is only recommended upon pulsatile tinnitus, a visual mass upon otoscopy, or a JT PGL on MRI [20]. In two patients, the localization of HNPGLs exhibited disparities between the two modalities, likely attributed to the anatomical nature of MRI and the more functional nature of the [^68^Ga]Ga-DOTATOC PET/CT. 

In this study, we accepted an interval of up to 12 months between performing an MRI and [^68^Ga]Ga-DOTATOC PET/CT because the natural growth rate of paragangliomas is generally limited, and in this study, it is the differences in the presence or absence of the tumor rather than the size that matter. The literature suggests an indolent tumor growth of 1.0 mm/year, but no updated research is available [21,22].

As our local protocol for wbMRI is less accurate compared to the dedicated protocol for the h-nMRI (including MRA), we suspect that relative smaller tumors will be missed. In our center, wbMRI is used for global screening for paraganglioma (vertex to groin), compared to the dedicated h-nMRI protocol, used when a patient is suspected of HNPGLs [20]. Therefore, JTPGLs will be most often undetected in a relatively early state. However, patients are included in a closely monitored follow-up schedule, and these tumors will be found upon tumor growth or the onset of symptoms.

There are additional considerations of [^68^Ga]Ga-DOTATOC PET/CT that warrant examination. The associated costs of [^68^Ga]Ga-DOTATOC PET/CT are twofold, compared to whole body MRI. Additionally, [^68^Ga]Ga-DOTATOC PET/CT exposes patients to a relatively high radiation dose of 2.1 mSv, equivalent to approximately 0.78 years of background radiation, using the Netherlands as reference with 2.8 mSv per inhabitant per year [23,24]. The scanning time of [^68^Ga]Ga-DOTATOC PET/CT is almost the same as h-nMRI, but the incubation period of approximately 45 min between the administration of the radiopharmaceutical and the initiation of the [^68^Ga]Ga-DOTATOC PET/CT acquisition may benefit patients with claustrophobia or anxiety disorders.

Twenty-four patients (96.0%) carried a variation in the succinate dehydrogenase complex, which causes different PGL syndromes. Over 40% of all PGL syndromes is caused by germline variants [4,25]. These syndromes exhibit clinical heterogeneity, making tumor behavior unpredictable. Multifocality is most prevalent in SDHD variation carriers (79%) compared to SDHB variation carriers (33%). Lifelong penetrance for the SDHB variation carriers is considerably lower than that of the paternally inherited SDHD variant [26,27]. Malignant tumors occur in 5% of SDHD variant carriers compared to 33% in SDHB variant carriers. SDHB variant carriers also have a relatively high mortality rate compared to SDHD variant carriers. Additional screening with whole body [^68^Ga]Ga-DOTATOC PET/CT in variation carriers at baseline can improve early tumor detection in a disease that can manifest throughout the body [28].

In one patient (#3 (*n* = 1/25, 4.0%)), the outcome of the [^68^Ga]Ga-DOTATOC PET/CT changed the treatment approach from initial surgical resection to a wait-and-scan policy. The patient, known to have a variant in SDHD and a surgical resection of a left- and right-sided CB PGLs in 1992, complicated by the paresis of the right vagus nerve, showed a recurrent right-sided CB PGL and left-sided JTPGL on the [^68^Ga]Ga-DOTATOC PET/CT compared to only a right-sided CB PGL on the h-nMRI. Therefore, the function of the left vagus nerve in particular will be closely monitored, to prevent permanent bilateral laryngeal failure.

[^68^Ga]Ga-DOTATOC PET/CT has other well-described indications, such as catecholamine hyper production and metastatic disease. A recent meta-analysis showed the superiority of [^68^Ga]GA-DOTATOC PET/CT over other functional imaging modalities, indicating further possibilities in detecting PGL(s) localizations as potential sources of hormone secretion and metastatic disease [15,29,30]. However, this pilot study did not focus on those aspects. 

The potential benefits of whole body PGL detection using [^68^Ga]Ga-DOTATOC PET/CT likely outweigh the disadvantages of increased costs and radiation dose. However, future cost-effectiveness analyses are needed to confirm this conclusion. 

Based on these findings, at the start of screening, specifically for carriers of a genetic variation associated with the development of paragangliomas, we consider performing only a [^68^Ga]Ga-DOTATOC PET/CT instead of MRI. If this scan is negative, it can be repeated after 3–5 years, with the timing depending in part on the age of the patient, any tumor-specific symptoms, and/or the increased excretion of catecholamines. Especially in SDHB variation carriers, the clinical utility of [^68^Ga]Ga-DOTATOC PET/CT can be significant, but more research is needed. An h-nMRI and/or CT of the mastoid bone could be used as follow-up according to the local protocols for the different genetic variations. Despite promising outcomes, [^68^Ga]Ga-DOTATOC PET/CT has not been implemented in the standard of care of the Netherlands yet, and a prospective study is needed to validate these results. 

## 5. Limitations

The limitations of this study include its retrospective design, selection bias, and limited sample size. 

## 6. Conclusions

Our study demonstrates the usefulness of [^68^Ga]Ga-DOTATOC PET/CT as an addition or initial replacement to the diagnostic armamentarium in patients with germline variants in SDHx. It provides whole-body screening without significantly reducing the diagnostic accuracy for HNPGLs. Used as the preferred baseline imaging modality, it can guide future imaging-based surveillance for HNPGLs. Prospective studies are needed to confirm these findings.

## Figures and Tables

**Table 1:** Characteristics of 25 patients with HNPGL who underwent MRI and [^68^Ga]Ga-DOTATOC PET/CT.

Baseline Characteristics		
Sex	No.	%
Female	14	56.0
Male	11	44.0
Variation	24	96.0
SDHA	2	8.0
SDHB	9	36.0
SDHC	1	4.0
SDHD	12	48.0
None	1	4.0
Catecholamine hyperproduction *	5	20.0
3-MT	3	
Metanephrines	0	
Normetanephrines	3	

3-MT = 3-methoxy tyramine; SDHA= Succinate Dehydrogenase A gene; SDHB = Succinate Dehydrogenase B gene; SDHC = Succinate Dehydrogenase C gene; SDHD = Succinate Dehydrogenase D gene. * One patient produced two types of hormones (3-MT + normetanephrine).

**Table 2:** Results of the wbMRI/h-nMRI compared to the [^68^Ga]Ga-DOTATOC PET/CT.

Pt. #	Age	Sex	Variation	BL/FU	MRI	[^68^Ga]Ga-DOTATOC PET/CT	SUVmax	Difference in Outcome
1	44	F	SDHD	FU	VPGL L&R *	CBPGL R + JPGL L	61.1 and 48.53	Different location
2	23	F	SDHB	FU	No lesions **	No lesions		-
3	52	M	SDHD	BL	VPGL R *	CBPGL R + JPGL L	49.3 and 31.2	CBPGL R
4	39	M	SDHB	BL	No lesions **	No lesions		-
5	46	M	SDHB	BL	No lesions *	No lesions		-
6	53	M	SDHD	FU	CBPGL L&R *	CB PGL L&R + JPGL R	40.4, 13.3 and 14.3	JPGL R
7	58	F	SDHD	BL	CBPGL L&R + VPGL R *	CB PGL L&R + VPGL R + TPGL L	155, 11.9, 166 and 8.6	TPGL L
8	31	F	SDHD	BL	CBPGL R *	CBPGL R	20.5	-
9	79	F	SDHC	FU	VPGL L&R + J/TPGL R **	VPGL L&R + JPGL R	12.1, 156 and 34.4	-
10	20	M	SDHB	FU	No lesions *	No lesions		-
11	44	F	SDHD	FU	JPGL L *	JPGL + VPGL R	17.5 and 7.3	VPGL R
12	26	M	SDHD	FU	No lesions **	CBPGL + TPGL R	40.9 and 2.7	CBPGL + TPGL R
13	51	F	SDHB	BL	No lesions **	No lesions		-
14	71	F	SDHD	FU	JPGL L + VPGL L *	CBPGL L + JPGL L	56.7 and 14.1	Different location
15	30	F	SDHB	BL	CBPGL L *	CBPGL L	47.7	-
16	28	M	SDHA	BL	CBPGL L *	CBPGL L	43.6	-
17	29	F	SDHB	BL	No lesions *	No lesions		-
18	32	F	SDHB	BL	No lesions *	No lesions		-
19	60	F	SDHD	FU	VPGL L&R *	VPGL L&R	169 and 64.7	-
20	60	M	SDHD	FU	No lesions *	TPGL L&R + VB	21.3 and 5.8	TPGL L&R + JPGL R
21	30	F	SDHD	FU	CBPGL R **	CBPGL R	23.4	-
22	40	M	SDHD	FU	CBPGL L&R+ VPGL R + JPGL L *	CBPGL L&R + VPGL R + T/JPGL L	17.6, 109, 133 and 78.7	-
23	70	F	SDHB	FU	T/JPGL L *	JPGL L	23.7	-
24	74	M	None	BL	CBPGL L *	CBPGL L	180	-
25	58	M	SDHA	FU	CBPGL L *	CBPGL L	25	-

[^68^Ga]Ga-DOTATOC PET/CT = somatostatin receptor positron emission computed tomography; BL = baseline; CB PGL = carotid body paraganglioma; F = female; FU = follow-up; JPGL = jugular paraganglioma; L = left-sided; M = male; MRI = magnetic resonance imaging; Pt = patient; R = right-sided; SDHA = Succinate Dehydrogenase A gene; SDHB = Succinate Dehydrogenase B gene; SDHC = Succinate Dehydrogenase C gene; SDHD = Succinate Dehydrogenase D gene; SUVmax = maximum standardized uptake value; TPGL = tympanic paraganglioma; VB = vestibular schwannoma; VPGL = vagal paraganglioma; * head-and-neck MRI = h-nMRI, ** whole body-MRI = wbMRI.

## Data Availability

Data are contained within the article.

## Appendix A

MRI Head and Neck Glomus sequence: skull base to clavicle: Axial (Ax) PD STIR, Ax T1 TSE, Ax DW SPIR, Coronal (Cor) T1 Gadolinium (Gd) MRA, Ax T1 DIXON Fat suppressed (FS) TSE Gd, Cor T1 Dixon TSE FS Gd.

MRI whole body paraganglioma sequence: skull base to groin: Sagittal (sg) survey, coronal (cor) survey, transversal (t) PD STIR head and neck, cPD STIR head and neck, T in- out phase thorax and abdomen, DWIBS, T2 SPAIR.
